# Altered microRNA composition in the uterine lumen fluid in cattle (*Bos taurus*) pregnancies initiated by artificial insemination or transfer of an in vitro produced embryo

**DOI:** 10.1186/s40104-024-01083-8

**Published:** 2024-09-13

**Authors:** Fernando H. Biase, Sarah E. Moorey, Julie G. Schnuelle, Soren Rodning, Martha Sofia Ortega, Thomas E. Spencer

**Affiliations:** 1 School of Animal Sciences, Virginia Polytechnic Institute and State University, 175 W Campus Dr, Blacksburg, VA 24061 USA; 2 Department of Animal Science, University of Tennessee, Knoxville, TN 37996 USA; 3 Department of Clinical Sciences, Auburn University, Auburn, AL 36849 USA; 4 Department of Animal Science, Auburn University, Auburn, AL 36849 USA; 5 Department of Animal and Dairy Sciences, University of Wisconsin Madison, Madison, WI 53706 USA; 6 Division of Animal Sciences, University of Missouri, Columbia, MO 65211 USA

**Keywords:** Artificial reproductive technology, Cattle, Conceptus, Embryos, Endometrium, Small RNA

## Abstract

**Background:**

MicroRNAs (miRNAs) are presented in the uterine lumen of many mammals, and in vitro experiments have determined that several miRNAs are important for the regulation of endometrial and trophoblast functions. Our aim was to identify and contrast the miRNAs present in extracellular vesicles (EVs) in the uterine lumen fluid (ULF) at the onset of attachment in cattle pregnancies (gestation d 18) initiated by artificial insemination (AI) or by the transfer of an in vitro-produced blastocyst (IVP-ET). A third group had no conceptus after the transfer of an IVP embryo.

**Results:**

The abundance of 263 annotated miRNAs was quantified in the EVs collected from ULF. There was an increase in the transcript abundance of 20 miRNAs in the ULF EVs from the AI pregnant group, while 4 miRNAs had a lower abundance relative to the group not containing a conceptus. Additionally, 4 miRNAs were more abundant in ULF EVs in the AI pregnant group relative to IVP-ET group (bta-mir-17, bta-mir-7-3, MIR7-1, MIR18A). Specific miRNAs in the ULF EVs were co-expressed with messenger RNAs expressed in extra-embryonic tissues and endometrium, including genes that are known to be their targets.

**Conclusions:**

The results provide biological insights into the participation of miRNAs in the regulation of trophoblast proliferation and differentiation, as well as in endometrium receptivity. The knowledge that in vitro cultured embryos can contribute to the altered abundance of specific miRNAs in the uterine lumen can lead to the development of corrective approaches to reduce conceptus losses during the first month of pregnancy in cattle.

**Supplementary Information:**

The online version contains supplementary material available at 10.1186/s40104-024-01083-8.

## Background

In cattle, the embryo enters the uterus by gestation d 4–5 and hatches from the zona pellucida by d 8 post-fertilization [1, 2]. At this time, the outer monolayer of trophectoderm cells and the uterine luminal epithelium (LE) have direct contact. The hatched blastocyst begins to produce interferon tau (IFNT) [3], which is the major pregnancy recognition signal that inhibits the development of the endometrial luteolytic mechanism [4, 5]. On gestation d 12–14, the blastocyst is ovoid in shape (~ 2–5 mm in length) and transitions into a tubular shape by d 14–15, at which time it can be termed a conceptus [6]. In cattle, elongation is also coincident with a greater release of IFNT [7]. The conceptus elongates via the proliferation of the trophectoderm and parietal endoderm cells [8] and reaches 20 cm or more in length by d 19–20 [8, 9]. Then, the trophectoderm begins to attach to the endometrial lining [8, 10] thereby initiating cell-to-cell communication mediated by adhesion [10]. By gestation d 25, a small proportion of trophectoderm cells have differentiated into binuclear cells [11], and the formation of the chorion marks the onset of the epitheliochorial placentation [1, 9, 12]. Ruminants have an epitheliochorial nature of placentation [1, 13, 14] with an extended time of non-invasive implantation followed by a limited invasion of the endometrium [10, 13].

After the primary signals or hormonal controllers exert their roles to progress endometrial and conceptus functionality, a myriad of cellular signals, mediated by bioactive molecules named embryotrophins [15], is triggered. The embryo can modulate the regulation of gene expression in the endometrium as early as on d 7 of gestation [16], when it can also promote alterations in the metabolite composition of the uterine lumen fluid (ULF) [17]. By gestation d 15–16, global alterations in endometrial gene expression occur in response to the elongating conceptus [18–20]. Thereafter, a synchrony of gene regulation between the extra-embryonic tissue (EET) and the endometrium is established [21] and contributes to a carefully orchestrated cell-to-cell communication between the conceptus and endometrium.

As the trophoblast and endometrium lining have direct contact, cell-to-cell interactions [10, 22, 23] can be established through ligand-receptor mediated signaling [20, 24, 25]. The transfer of RNAs from cell to cell is another concept [26], supporting the possible exchange of signals between trophoblast and endometrium [27]. These RNAs can be transported between cells in extracellular vesicles (EVs), which may also contain proteins and other macromolecules such as lipids [26, 28, 29]. EVs present in the ULF carrying microRNAs (miRNAs) have gained much attention in the past decade due to their importance in modulating trophoblast function and health [30] and endometrial remodeling [31]. Alterations in the miRNA content of EVs in the ULF have been associated with embryo implantation failure in women (reviewed in [32, 33]).

Cattle blastocysts [34–36], d 16 conceptuses [37] and endometrial cells [38, 39] produce EVs containing miRNAs. Alterations in miRNA profiles of EVs present in the ULF have been detected as early as pregnancy d 7 of gestation in cattle [40]. Also on d 7, blastocysts produced in vitro may produce and export miRNAs in EVs that are different from their in vivo generated counterparts [41]. EVs are present in the ULF during the attachment period [42–45] containing miRNAs among other molecules in their cargo [42, 43]. In vitro experiments have determined that the cargo in EVs present in the uterine lumen of pregnant cows can modulate gene transcription in cattle endometrial [42, 43, 45] and trophoblast [46] cell lines. However, the extent to which miRNAs present in the ULF contribute to the conceptus-maternal communication in cattle remains unclear. In the present study, our aims were to determine differences in the miRNA profiles in the ULF EVs of d 18 pregnancies harboring an in vivo derived conceptus versus ULF EVs when there is not conceptus present, and also to determine differences in the miRNA profiles in the ULF EVs of d 18 pregnancies harboring an in vivo or in vitro derived conceptus. Herein we tested two hypotheses: (a) that the miRNA profile in the ULF of d 18 pregnant uteri harboring in vivo conceptus is different than those of uteri harboring in vitro derived conceptus, and (b) that miRNAs can form co-expression regulatory networks with genes expressed in the conceptus and endometrium.

## Methods

All animal procedures for live handling were approved by the Institutional Animal Care and Use Committee, Auburn University, under protocol 2016-2874.

### In vitro production of embryos and cryopreservation

All chemicals were obtained from Sigma-Aldrich (St. Louis, MO, USA) or Fisher (Pittsburgh, PA, USA), unless otherwise stated. Embryo production procedures utilized in this study to produce embryos were consistent with procedures detailed previously [47–49]. Cumulus-oocyte complexes were aspirated from follicles (3–8 mm in diameter) of abattoir-derived ovaries. The cumulus-oocyte complexes were washed in Tissue Culture Medium-199 with Hanks salts supplemented with 25 mmol/L HEPES followed by in vitro maturation in Tissue Culture Medium-199 with Earle salts (Gibco, Grand Island, NY, USA) supplemented with 10% fetal bovine serum, 100 IU/mL penicillin, 100 μg/mL streptomycin, 0.2 mmol/L sodium pyruvate, 2 mmol/L L-glutamine, 50 ng/mL recombinant human epidermal growth factor (Invitrogen, Waltham, MA, USA), and 5 μg/mL of follicle-stimulating hormone (Bioniche Animal Health, Athens, GA, USA). In vitro maturation was carried out for 22–24 h at 38.5 °C in a humidified atmosphere containing 5% CO_2_.

In vitro matured cumulus-oocyte complexes were washed three times with HEPES-TALP medium and placed in IVF-TALP medium for in vitro fertilization. Sperm from a single sire was prepared by density gradient centrifugation utilizing the ISolate sperm separation kit (Irvine Scientific, Santa Ana, CA, USA) and washed twice by centrifugation in SP-TALP. Sperm was added to the fertilization dish at the concentration of 1 × 10^6^/mL, followed by the addition of penicillamine-hypotaurine-epinephrine solution. In vitro fertilization was carried out for 17–19 h at 38.5 °C in a humidified atmosphere containing 5% CO_2_. Cumulus cells were removed from putative zygotes by vortexing in 400 μL/of HEPES-TALP. Putative zygotes were then cultured in groups of up to 50 in 500 μL of SOF-BE2, covered with 300 μL of light mineral oil. In vitro culture was carried out at 38.5 °C in a humidified atmosphere containing 5% CO_2_, 5% O_2_ and 90% N_2_. Seven d after in vitro fertilization, grade one [50] blastocysts were cryopreserved using the slow-freezing procedure in ethylene-glycol solution [51].

### Estrous synchronization, artificial insemination, and embryo transfer

Nulliparous heifers of Angus-cross genetic background (15–19 months of age, weighing > 296 kg) were utilized for this experiment. Animals were randomized into one of the two experimental groups, based on whether they would be artificially inseminated or serve as recipients for embryo transfer.

Estrous synchronization [52] was initiated by inserting a controlled internal drug release (CIDR, 1.38 g progesterone), which was removed after 14 d. Sixteen d post removal of CIDR, 25 mg of prostaglandin F2 alpha (Lutalyse^®^, Zoetis, Parsippany-Troy Hills, NJ, USA) was administered along the application of an estrus detection patch (Estrotect™; Rockway Inc., Spring Valley, WI, USA) mid-way between the hip and tail head. All animals were observed for estrus by two investigators, and heifers that showed clear signs of standing estrus [53] and ≥ 50% of color change on the estrus detection patch continued the protocol. Alternatively, when there was no sign of estrus, the heifer was re-enrolled in estrous synchronization.

If the heifer was assigned to be artificially inseminated, AI was conducted 12–16 h after the onset of standing estrus. All heifers were inseminated with semen from one sire, which was the same sire used for in vitro embryo production.

For embryo transfer, 7 d post-estrus, the presence of a corpus luteum was evaluated by transrectal ultrasonography. If a corpus luteum was present, one embryo was deposited in the uterine horn ipsilateral to the corpus luteum. If the heifer did not present a corpus luteum, she was re-enrolled in estrous synchronization.

### Collection of the uterine lumen contents

Heifers were euthanized with captive bolt on d 18 of pregnancy (herein considered 18 d post fertilization). The reproductive tract was removed from each heifer within 15 min of euthanasia and immediately prepared for flushing. To prepare the reproductive tract for flushing, first, mesometrium was removed from the uterine horns. Next, the cervix was removed by cutting the base of the uterine body. Then, an 18-g needle coupled to a syringe was inserted into the distal portion of the ipsilateral horn and 20 mL of nuclease-free phosphate-buffered saline solution were flushed towards the base of the uterine body. The fluid was flushed into a cell strainer placed in a 50-mL conical tube, which retained a conceptus, when present, and clumps of cells. The flushed solution was centrifuged (3,000 × *g* for 15 min at 4 °C) for the palletization of cells and other debits. The supernatant was filtered using a polyvinylidene difluoride 0.45 μm membrane filters for the removal of potential remaining cells. The flushed material was stored at –80 °C until extraction of miRNAs.

**Fig. 1:**
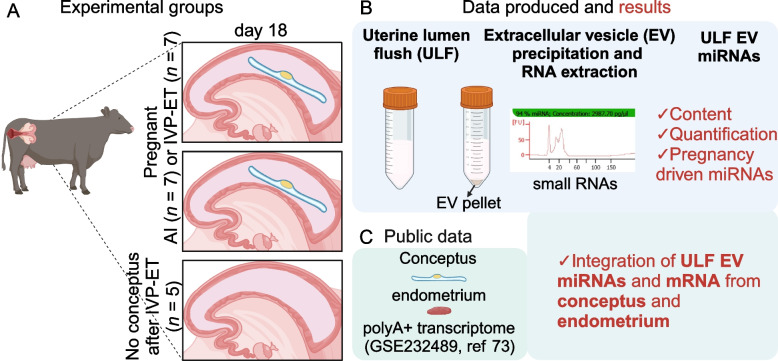
Overview of the experiment and study carried out. **A** Experimental groups included in the experiment. **B** Schematics of the procedures and data produced, as well as the results obtained. **C** Depiction of the data obtained from the public data base. Created with BioRender.com

Samples were categorized as obtained from pregnancies initiated by artificial insemination (AI, *n* = 7), or in vitro produced embryo followed by embryo transfer (IVP-ET, *n* = 7), or no conceptus present (NCP, *n* = 5) after embryo transfer (Fig. 1A).

### Processing of the uterine luminal flush and high throughput sequencing of small RNAs

Starting with 10 mL of ULF, EVs were captured and isolated using the ExoQuick-TC™ Tissue Culture Media Exosome Precipitation Solution (System Biosciences, Palo Alto, CA, USA) following the manufacturer’s protocol (Fig. 1B). The kit is based on the purification with polyethylene glycol [54] along with centrifugation, which is an acceptable method for the collection of EVs [55] and has been validated to precipitate EVs that are positive for the tetraspanins CD9 [56] or CD63 [57, 58] including in cattle follicular fluid [57]. Both markers are characteristic of EVs [55].

Particle size analysis was conducted in two representative samples (three technical replicates per sample) as a service provided by System Biosciences, Palo Alto, CA, USA. The size of EVs was assessed using a NanoSight NS300 instrument (NanoSight Ltd., Amesbury, UK) and a sCMOS camera, under shutter setting 1,000 and gain 400. The assays were conducted in parallel with standard beads (100 nm latex bead – standardized by the National Institute of Standards and Technology), which reported a 5% coefficient of variation in the measurements of the standard beads.

To further validate the enrichment of EVs, we separated two samples for precipitation of EVs as indicated above. We assayed EVs markers using the Exo-Check Exosome Antibody Arrays (System Biosciences, Palo Alto, CA, USA) according to the manufacturer’s manual. This slot blot has 12 pre-printed spots and out of which antibodies for exosome markers (CD63, CD81, ALIX, FLOT1, ICAM1, EpCam, ANXA5 and TSG101), and GM130, a *cis*-Golgi marker to identify cellular contamination. The signal was developed by chemiluminescence using WesternBright™ Sirius™ HRP substrate (Advansta, San Jose, CA, USA). The array was imaged on a iBright™ CL750 Imaging System (ThermoFisher Scientific).

After obtaining the pellet with EVs, small RNAs extracted using TRIzol Reagent [59] following procedures optimized for small sample size [60]. We accessed the small RNAs using the Agilent Small RNA kit (Agilent, Waldbronn, Germany) in a 2100 Bioanalyzer Instrument (Agilent) (Fig. 1B) and submitted the samples for sequencing at the VANTAGE (Vanderbilt Technologies for Advanced Genomics) at Vanderbilt University, Nashville, TN. Sequencing libraries were prepared with the NEBNext^®^ Small RNA Library Prep Set for Illumina^®^ (New England Biolabs, Ipswich, MA, USA) and sequencing was assayed in a NovaSeq 6000 Sequencing System (Illumina, San Diego, CA, USA) to produce a minimum of 10 million reads per sample (Fig. 1B).

Raw sequences were processed for the removal of adapters with Trimmomatic [61] and alignment to the bovine genome (Bos_taurus.ARS-UCD1.2.104) using bowtie2 (v.2.3.5.1) [62] using the “–very-sensitive” option. Next, Samtools (v 1.10) [63] was used to retain only primary alignments. Lastly, featurecounts (v 2.0.1) [64] was used to count reads according to the Ensemble annotation (Bos_taurus.ARS-UCD1.2.104) [65, 66]. Counts per million (CPM) was calculated with the function “cpm” from the “edgeR” package [67, 68] in R software. Lastly, only annotated small RNAs with > 50 sequences across all samples were retained for downstream analysis.

### Statistical analyses

Transcript abundance of miRNAs was compared using the R packages ‘edgeR’ [67, 68], with the quasi-likelihood test, and ‘DEseq2’ [69], using the Wald’s and likelihood test. The nominal *P* values of both tests were corrected for multiple hypothesis testing using the false discovery rate (FDR) method [70]. Differential transcript abundance was assumed when FDR < 0.05 for both tests.

Enrichment tests for Gene Ontology categories were carried out using “goseq” package [71] in R software. In all tests, the genes whose transcript abundances were estimated for the samples being tested were used as the background. The nominal *P* value was adjusted for multiple hypothesis testing by controlling the familywise error rate (FWER) following the method proposed by Holm [72] using the function “p.adjust” from the ‘stats’ R package. Significance was inferred when FWER ≤ 0.01.

Transcript abundance from mRNA data were obtained from EET and endometrium collected from the same uteri used to collect ULF EVs (GSE232489 [73], Fig. 1C). Co-expression analysis is an analytical approach to identify quantitative relationships between transcript abundances, and can be measured using a correlation coefficient [74]. Given the experimental design in our study, we were able to conduct a co-expression analysis between miRNAs and mRNAs for which we adopted the procedures recommended by Johnson and Krishnan [75]. All miRNAs and protein coding genes that passed our filtering for lowly expressed genes were used in this analysis. For each group and sample type (i.e., EET AI), CPM metric was calculated using the trimmed mean of M values method [76]. Then, CPM was transformed using the arcsine transformation $Log\left(x+ \sqrt{\left({x}^{2}+1\right)}\right)$ using the “asinh” function in R. Lastly, the Pearson coefficient of correlation and the corresponding *P* value were calculated using the function “corAndPvalue” from the package “WGCNA” [77] for each pair of genes (miRNA and mRNA). Empirical values of FDR (eFDR) [21, 78]were estimated with 10,000 randomizations of the data. Co-expression was inferred when eFDR < 0.00004 (equivalent to nominal *P* value < 0.00001).

### miRNA predicted targets

In order to assess if a specific protein coding genes would be a target of a specific miRNA, we obtained mRNA predicted targets for miRNAs from humans and cattle from the miRWalk database [79] (http://mirwalk.umm.uni-heidelberg.de/resources/) on November 26^th^ 2021, including untranslated regions and coding sequences.

## Results

**Fig. 2:**
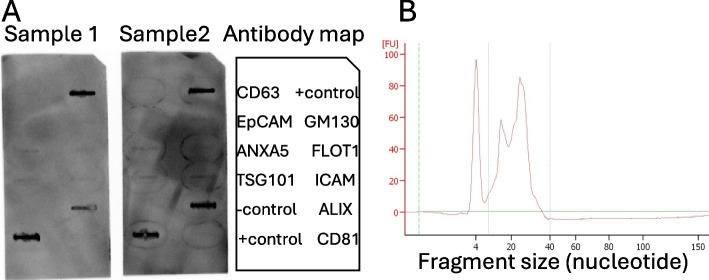
Overview of the biological samples. **A** Two unedited slot blots containing antibodies for EV markers, along positive and negative controls. **B** Representative image of the bioanalyzer assay for small RNAs

Following the nanoparticle tracking analysis with finite track length adjustment [80], the mode size distribution of the EV 90 and 97, with an average and standard deviation of the particle sizes of 148 ± 102 and 168 ± 120 nm, respectively. An assay of eight proteins that are common markers of EV showed a high abundance of ALIX in our EV preparation. Other proteins with signal but at a lower abundance were ANXA5, TSG101, ICAM and FLOT1. There were faint signals of CD63 and GM130 (Fig. 2A). Next we extracted RNAs from EVs out of which, most were miRNAs (Fig. 2B).

**Fig. 3:**
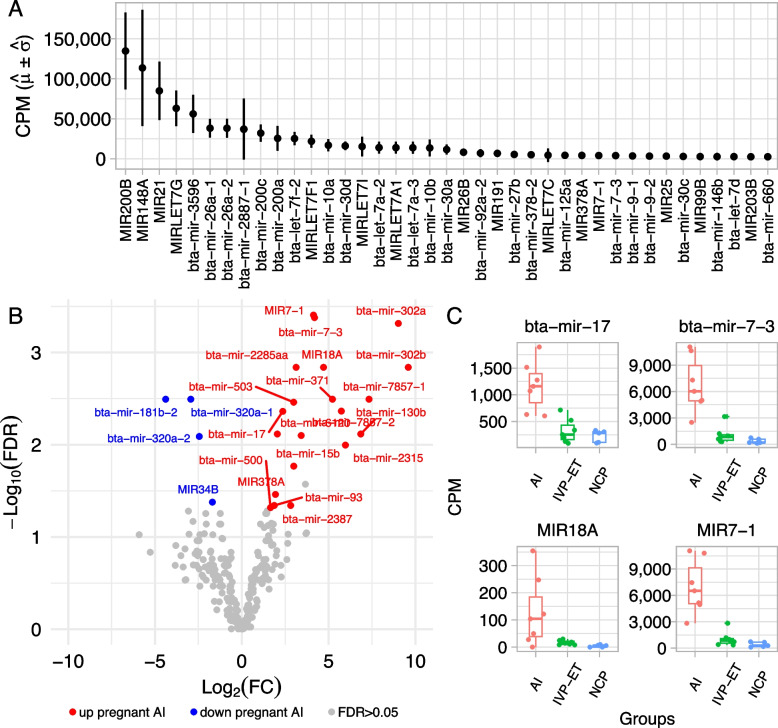
miRNAs in the ULF. **A** Top 40 most abundant miRNAs in the ULF EVs of pregnant heifers (AI group) on gestation d 18. **B** miRNAs with differential abundance in the ULF EVs of d 18 pregnant heifers (AI group) versus NCP counterparts. **C** miRNAs with differential abundance in the ULF EVs of pregnant heifers harboring an IVP-ET conceptus versus an AI conceptus. AI: artificial insemination; IVP-ET: in vitro produced and embryo transfer; NCP: no conceptus present after ET

Small RNA sequencing of EVs collected from the ULF was performed for 19 samples from gestation d 18 (AI, *n* = 7; IVP-ET, *n* = 7; NCP, *n* = 5). Altogether, over 537 million reads were produced from the small RNAs obtained from the EVs in the ULF, out of which 9,082,232 sequences matched to miRNAs on the Ensembl annotation averaging of 478,012 sequences per samples. After filtering for annotated miRNAs that had more than 50 reads across all samples, there were 263 annotated miRNAs (Additional file 1). Notably, the top 40 miRNAs present in the ULF flushed from pregnant uterus on d 18 of gestation accounted for 92% of the total reads produced and mapped to annotated miRNAs (Fig. 3A).

There were 24 miRNAs with differential abundance in the EVs obtained from ULF in the AI group versus the NCP group (greater abundance in AI: bta-mir-130b, bta-mir-15b, bta-mir-17, bta-mir-2285aa, bta-mir-2315, bta-mir-2387, bta-mir-302a, bta-mir-302b, bta-mir-371, bta-mir-500, bta-mir-503, bta-mir-6120, bta-mir-7-3, bta-mir-7857-1, bta-mir-7857-2, bta-mir-93, MIR18A, MIR378A, MIR7-1; lower abundance in AI: bta-mir-181b-2, bta-mir-320a-1, bta-mir-320a-2, MIR34B, FDR < 0.05, Fig. 3B, Additional file 2). Four miRNAs were more abundant in the EVs obtained from ULF of the AI group (bta-mir-17, bta-mir-7-3, MIR18A, MIR7-1, FDR < 0.05, Fig. 3C, Additional file 3) relative to those in the IVP-ET group.

Because miRNAs mostly degrade mRNAs, we tested whether the four miRNAs with higher abundance in the ULF EVs from AI group as compared to IVP-ET group could contribute to lower abundance of target mRNAs in conceptus and endometrial tissues collected from the same uteri (GSE232489 [73]). According to target predictions for humans and cattle present in the miRWalk database [79], the mature forms of bta-mir-17, bta-mir-7-3, MIR18A, MIR7-1, could be targeting 116, 397, 453 genes that have lower abundance in extra-embryonic tissues, caruncular and intercaruncular areas of the endometrium, respectively. Although there was no significant enrichment, the biological functions with the greatest number of genes with reduced transcript abundance in extra-embryonic tissues were “signal transduction” (5 genes), “regulation of transcription by RNA polymerase II” (5 genes), and “transmembrane transport” (4 genes) (Additional file 4). In the caruncular areas of the endometrium, most genes were annotated with “regulation of DNA-templated transcription” (32 genes), “signal transduction” (27 genes), and “protein phosphorylation” (25 genes) (Additional file 5). By contrast, the biological process “cilium movement” (7 genes) was significantly enriched (FWER = 0.0787) for intercaruncular areas of the endometrium. The categories with the greatest number of genes were “regulation of transcription by RNA polymerase II” (23 genes), “protein phosphorylation” (18 genes), and “transmembrane transport” (16 genes) (Additional file 6).

**Fig. 4:**
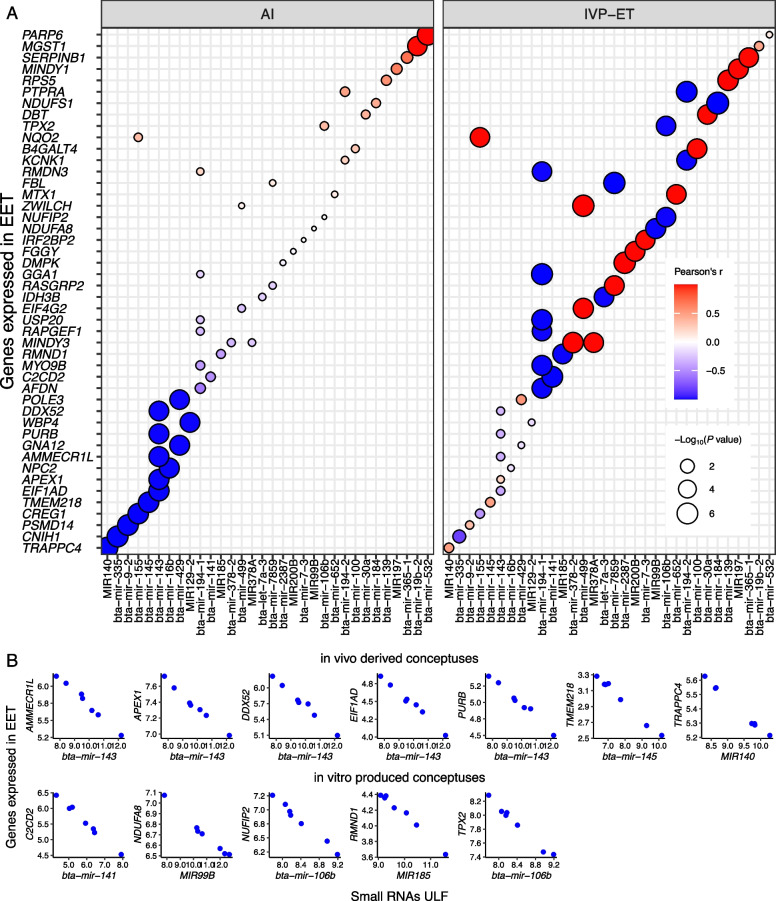
Coexpression between protein-coding genes expressed in d 18 conceptuses and miRNAs in the surrounding ULF EVs. Only genes annotated with a symbol are depicted in the figure. AI: artificial insemination, IVP-ET: in vitro produced and embryo transfer

We also interrogated the data to understand whether miRNAs present in the ULF of d 18 pregnant heifers would have a co-expression pattern with genes expressed in the conceptus. Our analysis showed that 20 genes (*AMMECR1L*, *APEX1*, *CNIH1*, *CREG1*, *DDX52*, *EIF1AD*, *GNA12*, *MGST1*, *NPC2*, *PARP6*, *POLE3*, *PSMD14*, *PURB*, *TMEM218*, *TRAPPC4*, *WBP4*, etc.) had transcripts co-expressing with 15 miRNAs in the ULF of heifers pregnant by AI (bta-mir-143, bta-mir-145, bta-mir-155, bta-mir-16b, bta-mir-19b-2, bta-mir-335, bta-mir-429, bta-mir-532, bta-mir-9-2, MIR129-2, MIR140, etc.) (FDR < 0.0004, Fig. 4A, Additional file 7). Notably, the co-expressing pairs of protein-coding genes (*n* = 36) and miRNAs (*n* = 27) present in the ULF of heifers harboring a conceptus produced in vitro were different from those identified in pregnancies initiated by AI (genes: *AFDN*, *B4GALT4*, *C2CD2*, *DBT*, *DMPK*, *EIF4G2*, *FBL*, *FGGY*, *GGA1*, *IDH3B*, *IRF2BP2*, *KCNK1*, *MINDY1*, *MINDY3*, *MTX1*, *MYO9B*, *NDUFA8*, *NDUFS1*, *NQO2*, *NUFIP2*, *PTPRA*, *RAPGEF1*, *RASGRP2*, *RMDN3*, *RMND1*, *RPS5*, *SERPINB1*, *TPX2*, *USP20*, *ZWILCH*; miRNAs: bta-let-7a-3, bta-mir-100, bta-mir-106b, bta-mir-139, bta-mir-141, bta-mir-155, bta-mir-184, bta-mir-194-1, bta-mir-194-2, bta-mir-2387, bta-mir-30a, bta-mir-365-1, bta-mir-378-2, bta-mir-499, bta-mir-652, bta-mir-7-3, bta-mir-7859, MIR185, MIR197, MIR200B, MIR378A, MIR99B, FDR < 0.0004, Fig. 4A, Additional file 7). Twelve pairs of miRNAs (7 in the AI group and 5 in the IVP-ET group) and protein-coding genes with inverted correlation were supported by the miRWalk database [79] of miRNA targets (Fig. 4B).

**Fig. 5:**
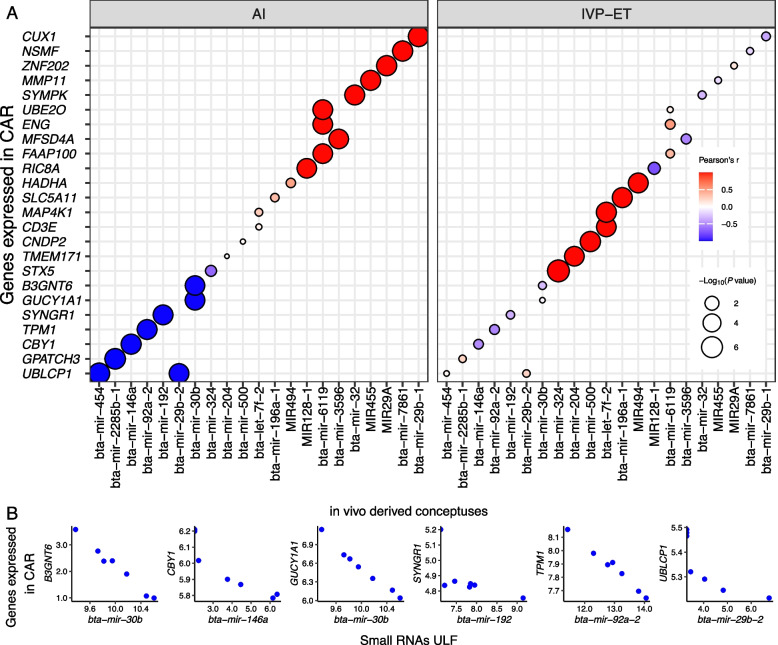
Coexpression between protein-coding genes expressed in caruncular (CAR) areas of the endometrium on gestation d 18 and miRNAs in the ULF EVs. Only genes annotated with a symbol are depicted in the figure. AI: artificial insemination, IVP-ET: in vitro produced and embryo transfer

We also identified co-expressing pairs between protein-coding genes expressed in the endometrium and miRNAs present in the ULF. In caruncular areas of the endometrium of pregnancies harboring a conceptus produced by AI, there were 20 genes (*B3GNT6*, *CBY1*, *CUX1*, *ENG*, *FAAP100*, *GPATCH3*, *GUCY1A1*, *MFSD4A*, *MMP11*, *NSMF*, *RIC8A*, *SYMPK*, *SYNGR1*, *TPM1*, *UBE2O*, *UBLCP1*, *ZNF202*, etc.) forming co-expression pairs with 16 miRNAs (bta-mir-146a, bta-mir-192, bta-mir-204, bta-mir-2285b-1, bta-mir-29b-1, bta-mir-29b-2, bta-mir-30b, bta-mir-32, bta-mir-3596, bta-mir-454, bta-mir-6119, bta-mir-7861, bta-mir-92a-2, MIR128-1, MIR29A, MIR455) (FDR < 0.0004, Fig. 5A, Additional file 8). By contrast, only 8 genes (*CD3E*, *CNDP2*, *HADHA*, *MAP4K1*, *SLC5A11*, *STX5*, *TMEM171*, etc.) formed co-expression with 6 miRNAs (bta-let-7f-2, bta-mir-196a-1, bta-mir-204, bta-mir-324, bta-mir-500, MIR494) in pregnancies initiated by the transfer of an in vitro-produced embryo (FDR < 0.0004, Fig. 5A, Additional file 8). Only six pairs of miRNAs and protein-coding genes with inverted correlation in pregnancies initiated by AI were supported by the miRWalk database [79] of miRNA targets (Fig. 5B).

**Fig. 6:**
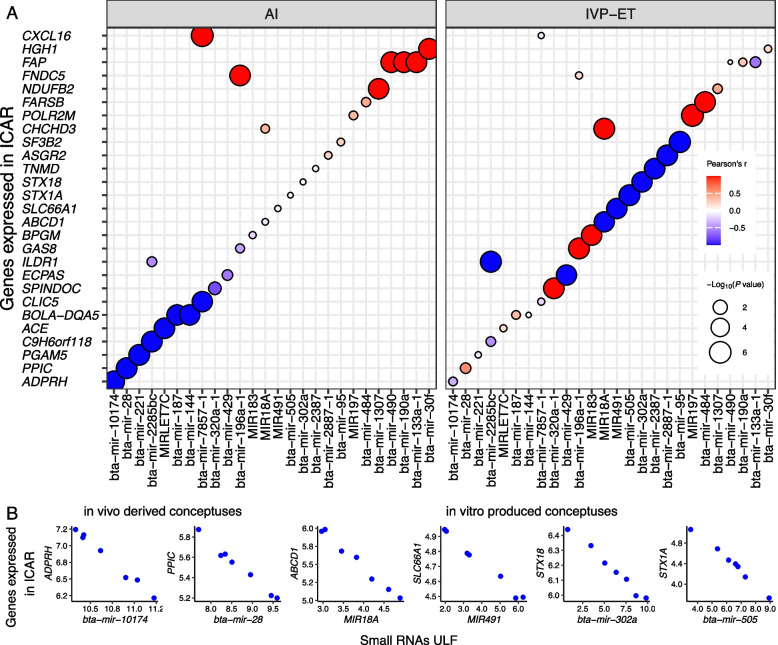
Coexpression between protein-coding genes expressed in inter-caruncular (ICAR) areas of the endometrium on gestation day and miRNAs in the ULF. Only genes annotated with a symbol are depicted in the figure. AI: artificial insemination, ET: embryo transfer

In intercaruncular areas of the endometrium, there were 16 genes (*A2M*, *ACE*, *ADPRH*, *BOLA*-*DQA5*, *C25H16orf71*, *C9H6orf118*, *CLIC5*, *CXCL16*, *FAP*, *FNDC5*, *HENMT1*, *HGH1*, *NDUFB2*, *PGAM5*, *PPIC*, *TSPAN17*) forming co-expression pairs with 18 miRNAs (bta-mir-10174, bta-mir-1307, bta-mir-133a-1, bta-mir-144, bta-mir-187, bta-mir-190a, bta-mir-196a-1, bta-mir-221, bta-mir-2285bc, bta-mir-28, bta-mir-30f, bta-mir-490, bta-mir-7857-1, MIRLET7C, etc.) (FDR < 0.0004, Fig. 6A, Additional file 9). By contrast, only 17 genes (*ABCD1*, *ASGR2*, *BPGM*, *CHCHD3*, *ECPAS*, *FARSB*, *GAS8*, *ILDR1*, *POLR2M*, *SF3B2*, *SLC66A1*, *SPINDOC*, *STX18*, *STX1A*, *TNMD*, etc.) formed co-expression with 17 miRNAs (bta-mir-196a-1, bta-mir-2285bc, bta-mir-2387, bta-mir-2887-1, bta-mir-302a, bta-mir-320a-1, bta-mir-429, bta-mir-484, bta-mir-505, bta-mir-95, MIR183, MIR18A, MIR197, MIR200B, MIR491, etc.) in pregnancies initiated by the transfer of an in vitro-produced embryo (FDR < 0.0004, Fig. 6A, Additional file 9). We identified six pairs of miRNAs and protein-coding genes with inverted correlation supported by the database of miRNA targets (Fig. 6B).

## Discussion

The ULF contains EVs during the cycle and early pregnancy in all studied mammals including cattle [27, 81–84]. The ULF EVs contain small RNAs, and their contents change throughout pregnancy [42–45]. Trophoblast and epithelial cells in the endometrium can uptake these EVs and their content [42, 43, 45, 46], supporting the idea that small RNAs present in the ULF have a role in pregnancy establishment. The particle size analysis confirmed that the purification with polyethylene glycol [54] enriched the pellet with EVs (30–150 nm [85], 30–200 nm [86]), while avoiding apoptotic bodies or cells. The protein ALIX has been identified in EVs isolated from conceptuses [37, 41], but this result does not eliminate that those EVs could also have come from the endometrium. Notably, however, is that ALIX may have a role into enriching miRNAs into EVs during their biogenesis [87]. Collectively, most, if not all, of miRNAs reported in this study were present in EVs originated from the conceptus and endometrium.

Our study has shortcomings. First our experimental design did not allow us to identify the origin of the EVs, hence the discussion addresses miRNAs present in EVs in the ULF without attempting to sort out their origin. Second, we did not measure progesterone nor IFNT, which are important elements in the establishment of pregnancy. Third, we did not carry out specific mechanistic studies to evaluate the impact of disturbing miRNAs on conceptuses or endometrium nor corresponding cell lines. Such shortcomings hinder the establishment of causation [88] and origin of the EVs. However, the careful hypothesis-driven data analysis of a rich and unique dataset overlapped with a reputable database that pairs miRNA and their targets provided important biological insights into the importance of miRNA cargo in EVs present in the ULF.

Our comprehensive analysis of miRNAs in the ULF of cyclic and pregnant heifers on gestation d 18 confirms that miRNA profiles in the ULF change in consequence of pregnancy. Four miRNAs were downregulated in the ULF on gestation d 18, and there is a compelling body of evidence supporting that the downregulation of these miRNAs is necessary for appropriate attachment of the conceptus to the endometrium. In rats, the abundance of mir-320 was lower on gestation d 5 in the endometrium [89]. The hsa-miR-320a is two-fold more abundant in the ULF of women with recurrent implantation failure compared to healthy fertile women [90]. The upregulation of mir-320a inhibits the growth and invasion of human extravillous trophoblast cell line HTR-8/SVneo by targeting interleukin 4 [91]. Also in humans, the upregulation of miR-34b in the endometrium is associated recurrent implantation failure [92]. In vitro experiments indicate that miR-34b inhibits cell proliferation by targeting Wnt/β-catenin [93] or Notch 1 [94] signaling pathways. In mice, the entire mir-181 family members, including mir-181b, which targets Leukemia Inhibitory Factor mRNA, were downregulated in the uterus on d 4 of pregnancy [95]. Taken together, the lower abundance of the miRNAs bta-mir-181b-2, bta-mir-320a-1, bta-mir-320a-2, and miR-34B in the uterine lumen creates an environment that is permissive to trophoblast proliferation and differentiation, which are essential for conceptus development during attachment.

Twenty miRNAs had greater transcript abundance in the ULF of a pregnant uterus relative to the non-pregnant counterparts. The bta-mir-302a and bta-mir-302b were among the topmost significant miRNAs, with greater fold change in pregnant versus not-pregnant uterus. mir-302a-3p was detected in porcine trophoblast but not in the endometrium [96], thus it is possible that the trophectoderm is the source of this miRNA in the ULF. miR-302 can induce and maintain pluripotency in trophoblast cells [97], and its expression is reduced upon pharmacological induction of differentiation of trophoblast cells [98]. Also of note, miR-15b is one of the three miRNAs that can stimulate the differentiation of mouse embryonic stem cells into trophoblast-like cells and sustain self-renewal properties [97]. In humans, miR-15b-5p stimulates trophoblast cell growth and migration [99]. The miRNA miR-503 is also highly expressed in mouse-differentiated trophoblast cells [100]. Collectively, these miRNAs with greater abundance in the ULF on gestation d 18 of pregnancy may have a role in maintaining the balance of stemness and differentiation of the trophoblast cells.

Four miRNAs were upregulated in the ULF of the pregnant uterus and were also down-regulated in the ULF when conceptuses were produced in vitro (mir-7-1, mir-7-3, mir-17, mir-18a). Interestingly, mir-7 downregulates the TGF-β-SMAD family member 2 pathway [101], promoting proliferating extravillous trophoblast cells [102, 103]. mir-17 inhibits trophoblast differentiation by regulating *hGCM1* and *hCYP19A1 *[104]. miR-18a was detected in both placental trophoblasts and endothelial cells [105–107] and promotes trophoblast cell differentiation [30] and invasion [105, 108, 109]. Collectively, these data would suggest that mir-7-1, mir-7-3, mir-17, mir-18a have an important role in the balance between proliferation and differentiation of trophoblast cells. It is important to note, that the lower abundance of mir-7-1, mir-7-3, mir-17, mir-18a in the ULF of pregnancies initiated by the transfer of an in vitro produced conceptus may be impactful to the differentiation of binucleate cells, which are less abundant in the chorion of some pregnancies harboring an in vitro produced conceptus [110].

Most miRNAs execute their roles by promoting mRNA degradation and/or inhibiting translation [111, 112]. To that end, we analyzed our dataset in conjunction with mRNA transcriptome obtained from the same reproductive tracts [73] to investigate co-expression between miRNA and mRNAs. The inverted co-expression, inferred from our data, of predicted miRNA—mRNA target pairs, from mirWalk database, support the hypothesis that miRNAs present in the ULF regulate transcript abundance of protein-coding genes in the conceptus and endometrium during the early stages of pregnancy. This could be explained by exchange of miRNAs between cells [29] where miRNAs can be enclosed into EVs [113], exported to the ULF and be up taken by either the conceptus or endometrium, thus a potential mechanism of signaling between conceptus and endometrium. While we could not determine the origin of the miRNA, co-expression analysis provided clues about the tissue where they are exerting their action.

The results also showed that the co-expression between miRNAs in the ULF and mRNAs (EET and endometrium) were remarkably different based on whether the conceptus was produced in vitro or generated by AI. Because the origin of the miRNAs was not determined in our experiments, we cannot determine whether those differences were caused by the conceptus or by the endometrial differential biosensing [25, 114, 115] of the conceptus’ origin. However, this differential co-expression adds another layer of complexity to the myriad of individualized [21] molecular interaction between conceptus and endometrium at attachment.

Notably, mir-143 showed co-expression with multiple target genes in EET obtained from pregnancies initiated by AI. In pigs, mir-143-3p is present in the luminal fluid and is taken by trophoblast cells promoting cell proliferation and migration [116]. Although there was no co-expression between mir-143 and genes expressed in the endometrium in our analysis, mir-143 may also have a role in endometrial cells. In mice, mir-143 is highly expressed in the subluminal stroma at implantation sites [117]. In rats, endometrial cells express mir-143 on gestation d 5–8, and experiments carried out in human endometrial stromal cells showed that mir-143 inhibits cell proliferation, migration, and invasion [118]. In women, mir-143 expression in the endometrial epithelium is induced by progesterone, and mir-143 inhibits the proliferation of endometrial cancer cells [119]. Among the co-expression pattern inferred in the EET of pregnancies with an in vitro produced conceptus, miR-141 is also expressed in trophoblast cells, and can regulate trophoblastic cell viability and proliferation [120].

One interesting observation about the co-expression between miRNA and mRNAs in the endometrium is that there were less pairs of co-expressing genes relative to the EET. Second, the co-expressing pairs detected in caruncular and inter-caruncular areas of the endometrium were different, which is not a surprising finding because these areas have genes with differential transcript abundance [25], reflecting their anatomical and physiological differences [10, 23, 121, 122]. Notably, mir-30b had a co-expression pattern with two genes (*B3GNT6*, *GUCY1A1*). In women, mir-30b [123, 124] and mir-30d [123] (14^th^ most abundant miRNA in the ULF) may participate in the regulation of endometrial receptivity, although the functional mechanisms remain unknown. The miRNA mir-28, which formed a co-expression pattern with *PPIC* in inter-caruncular areas of the endometrium, had greater transcript abundance in implantation sites relative to inter-implantation sites in mice on d 5 of pregnancy [125]. Also, in the inter-caruncular area of the endometrium, MIR18A showed a co-expression with *ABCD1*, revealing a potential role of this miRNA in the endometrium. It is also notable that there was a greater transcript abundance of this miRNA in the ULF of pregnancies initiated by AI relative to non-pregnant or pregnancies initiated by the transfer of an in vitro cultured embryo.

The results of co-expression between miRNAs in the ULF and mRNAs of target genes is a strong indication of the participation of those miRNAs in the ULF in the regulation of transcript abundance in EET and endometrium. However, it is important to highlight that our experiment did not determine the impact of that regulation on the conceptus, endometrium, or pregnancy health. For instance, the excessive abundance of mir-143 [90] and mir-145 [126, 127] have been associated with pregnancy failure in women and mice. A high abundance of mir-29b has been associated with preeclampsia in women [128], and the ULF of women with recurrent implantation failure had 2.7-fold more transcripts of mir-491 relative to the ULF of healthy fertile women [90].

## Conclusions

In summary, the results of this study support the idea that EVs present in the ULF of cows on d 18 of gestation contain a myriad of miRNAs. The alteration in the abundance of specific miRNAs in pregnant uterus versus non-pregnant ones indicates a role of specific miRNAs in the regulation of trophoblast health, endometrial remodeling, and the establishment of pregnancy. The presence of a conceptus produced in vitro was associated with the failure to increase the abundance of 4 miRNAs and suggests another layer of complexity that contributes to the lower success of pregnancy establishment of in vitro-produced embryos.

## Supplementary Information

**Additional file 1.** Annotated miRNAs present in the uterine luminal fluid.

**Additional file 2.** Analysis of differential abundance of miRNA in the EVs obtained from uterine luminal fluid in the AI pregnant group versus the 'no conceptus present' group.

**Additional file 3.** Analysis of differential abundance of miRNA in the EVs obtained from uterine luminal fluid in the AI pregnant group versus the ET pregnant group.

**Additional file 4.** Annotation of biological processes of the genes that can be targets of bta-mir-17, bta-mir-7-3, MIR18A, MIR7-1 and are also down regulated in extra-embryonic tissues of pregnancies initiated by artificial insemination versus pregnancies initiated by the transfer of an in vitro produced embryo.

**Additional file 5**. Annotation of biological processes of the genes that can be targets of bta-mir-17, bta-mir-7-3, MIR18A, MIR7-1 and are also down regulated in caruncular areas of endometrium of pregnancies initiated by artificial insemination versus pregnancies initiated by the transfer of an in vitro produced embryo.

**Additional file 6**. Annotation of biological processes of the genes that can be targets of bta-mir-17, bta-mir-7-3, MIR18A, MIR7-1 and are also down regulated in inter-caruncular areas of endometrium of pregnancies initiated by artificial insemination versus pregnancies initiated by the transfer of an in vitro produced embryo.

**Additional file 7**. Coexpression between miRNAs in the uterine luminal fluid and the genes expressed in extra-embryonic tissue.

**Additional file 8**. Coexpression between miRNAs in the uterine luminal fluid and the genes expressed in caruncular areas of the endometrium.

**Additional file 9**. Coexpression between miRNAs in the uterine luminal fluid and the genes expressed in intercaruncular areas of the endometrium.

## Data Availability

The datasets supporting the conclusions of this article are available in the Gene Omnibus Repository under the identifier GSE232489.

## Abbreviations

AI: Artificial insemination
bta: *Bos taurus*
CIDR: Controlled intravaginal drug release
CPM: Counts per million
EET: Extra-embryonic tissue
eFDR: Empirical false discovery rate
ET: Embryo transfer
EV: Extracellular vesicles
FDR: False discovery rate
FWER: Family wise error rate
HEPES: 4-(2-Hydroxyethyl)-1-piperazine ethanesulfonic acid
IFNT: Interferon tau
IVF: In vitro fertilization
IVP: In vitro produced
IU: International units
LE: Luminal epithelium
miRNA: MicroRNA
mRNA: Messenger ribonucleic acid
*n*: Sample size
NCP: No conceptus present
*P*: Probability
sCMOS: Scientific Complementary Metal–Oxide–Semiconductor
TALP: Tyrode’s albumin lactate pyruvate
ULF: Uterine lumen fluid
